# The impact of different first-line EGFR-TKIs on the clinical outcome of sequential osimertinib treatment in advanced NSCLC with secondary T790M

**DOI:** 10.1038/s41598-021-91657-7

**Published:** 2021-06-08

**Authors:** Yen-Hsiang Huang, Jeng-Sen Tseng, Kuo-Hsuan Hsu, Kun-Chieh Chen, Kang-Yi Su, Sung-Liang Yu, Jeremy J. W. Chen, Tsung-Ying Yang, Gee-Chen Chang

**Affiliations:** 1 Division of Chest Medicine, Department of Internal Medicine, Taichung Veterans General Hospital, No. 1650, Sect. 4, Taiwan Boulevard, Taichung, 407 Taiwan; 2 Institute of Biomedical Sciences, National Chung Hsing University, No. 145, Xingda Rd., South Dist., Taichung, 402 Taiwan; 3 Faculty of Medicine, School of Medicine, National Yang-Ming University, No. 155, Sect. 2, Linong St., Taipei, 112 Taiwan; 4 Division of Critical Care and Respiratory Therapy, Department of Internal Medicine, Taichung Veterans General Hospital, No. 1650, Sect. 4, Taiwan Boulevard, Taichung, 407 Taiwan; 5 Division of Pulmonary Medicine, Department of Internal Medicine, Chung Shan Medical University Hospital, No.110, Sec. 1, Jianguo N. Road, Taichung, 402 Taiwan; 6 Institute of Medicine, Chung Shan Medical University, No.110, Sec. 1, Jianguo N. Road, Taichung, 402 Taiwan; 7 School of Medicine, Chung Shan Medical University, No. 110, Sec. 1, Jianguo N. Road, Taichung, 402 Taiwan; 8 Department of Clinical Laboratory Sciences and Medical Biotechnology, College of Medicine, National Taiwan University, No. 1, Sect. 1, Jen Ai Road, Taipei, 100 Taiwan; 9 Department of Laboratory Medicine, National Taiwan University Hospital, No. 7, Zhung-Shan South Road, Taipei, 100 Taiwan; 10 Graduate Institute of Clinical Medicine, College of Medicine, National Taiwan University, No. 1, Sect. 1, Jen Ai Road, Taipei, 100 Taiwan; 11 Center of Genomic and Precision Medicine, National Taiwan University, No. 2, Syu-jhou Road, Taipei, 100 Taiwan; 12 Institute of Medical Device and Imaging, College of Medicine, National Taiwan University, No. 1, Sect. 1, Jen Ai Road, Taipei, 100 Taiwan; 13 Graduate Institute of Pathology, College of Medicine, National Taiwan University, No. 1, Sect. 1, Jen Ai Road, Taipei, 100 Taiwan

**Keywords:** Cancer therapy, Lung cancer

## Abstract

The impact of different first-line epidermal growth factor receptor (EGFR)-tyrosine kinase inhibitor (TKI)s to the clinical efficacy of osimertinib in *EGFR*-mutant non-small-cell lung cancer (NSCLC) patients with acquired T790M was still unclear. We enrolled 733 advanced *EGFR*-mutant NSCLC patients with gefitinib, erlotinib or afatinib as first-line EGFR-TKIs treatment for analysis. 373 patients received re-biopsies after progressive disease to first-line EGFR-TKIs treatment, and the total positive rate of T790M was 51.7%. 151 patients who harbored T790M received osimertinib as subsequent treatment. Among them, the median progression-free survival (PFS) of first-line EGFR-TKI (PFS1) was 14.0 months, and the median PFS of osimertinib (PFS2) was 10.1 months. The median PFS1 + PFS2 was 27.5 months, and the median overall survival from first-line EGFR-TKI was 61.3 months. Concerning different first-line EGFR-TKIs, the median PFS2 was 10.9 months in the gefitinib group, 10.0 months in the erlotinib group, and 6.7 months in the afatinib group (p = 0.534). The median PFS1 + PFS2 was 27.7 months, 26.8 months and 24.0 months in the gefitinib, erlotinib, and afatinib group, respectively (p = 0.575). In conclusion, both first-generation and second-generation EGFR-TKIs sequential osimertinib treatment provided good clinical efficacy in advanced *EGFR*-mutant NSCLC patients with acquired T790M mutation.

## Introduction

Non-small-cell Lung Cancer (NSCLC) accounts for 80–85% of the patients with lung cancer^1^. The treatment for NSCLC is found in the era of precision medicine. The therapeutic option is individualized and based on the results of histology and molecular biology tests. Patients diagnosed with oncogenic driver mutation will experience better overall survival if they received genotype-directed therapy^2^. *Epidermal growth factor receptor (EGFR)* mutation is the most common driver mutation gene amongst Asian patients with advanced NSCLC^3^. Approximately 50–60% of NSCLC patients in Asia have *EGFR* mutation^3,4^, while only 10–20% of patients in the western world experience it^2,5^. In 2004, Lynch et al*.* found that specific mutations in the *EGFR* gene were correlated with a clinical response to EGFR-tyrosine kinase inhibitors (TKI)^6^. Since that time, several clinical trials have proved that *EGFR*-mutant advanced NSCLC patients with first-generation (gefitinib and erlotinib) and second-generation (afatinib) EGFR-TKI treatment experienced longer progression-free survival (PFS) and fewer adverse effects than those patients who underwent platinum-based chemotherapy^7–9^.

Concerning different generation EGFR-TKIs, clinical trials of LUX-Lung 7 and ARCHER-1050 both showed second-generation (afatinib and dacomitinib) EGFR-TKIs significantly improved PFS more so than first-generation (gefitinib) EGFR-TKIs in *EGFR*-mutant NSCLC patients^10,11^. In ARCHER-1050 study, better Overall Survival (OS) was found in patients without brain metastasis. Additionally, the FLAURA study presented the third-generation EGFR-TKI, osimertinib, which displayed better PFS and OS than first-generation EGFR-TKIs in NSCLC patients with EGFR mutation^12,13^.

However, according to previous clinical trials, most of patients had acquired resistance 8 to 13 months later after first-line, first- and second-generation EGFR-TKIs use. Among the various mechanisms showing acquired resistance to first- and second-generation EGFR-TKIs, the secondary *EGFR* mutation, T790M mutation, accounted for 50–60% of the resistance mechanisms^14^. Our previous study had demonstrated that baseline *EGFR* exon 19 deletion and longer PFS of first-line EGFR-TKIs were both correlated with a higher frequency of the T790M mutation^15^. Fortunately, the third-generation EGFR-TKI can overcome T790M mutation, and provide significantly longer PFS than standard platinum-based chemotherapy in advanced T790M-positive NSCLC patients who had acquired resistance to first-line EGFR-TKI treatment^16^.

Although there are several real-world pieces of data which outline the difference in clinical efficacy between first- and second-generation EGFR-TKIs in advanced EGFR-mutant NSCLC patients, the results were inconsistent. Additionally, previous studies have been published to show the real-world effectiveness of osimertinib in T790M-positive NSCLC patients with an acquired resistance to EGFR-TKIs. However, few papers have focused on the impact of different first-line EGFR-TKIs to the clinical outcomes of sequential osimertinib treatment in patients with secondary T790M mutation. Therefore, we conducted the present study in order to investigate the important issues mentioned above.

## Results

### Patient characteristics for first-line gefitinib, erlotinib and afatinib treatment

**Figure 1:**
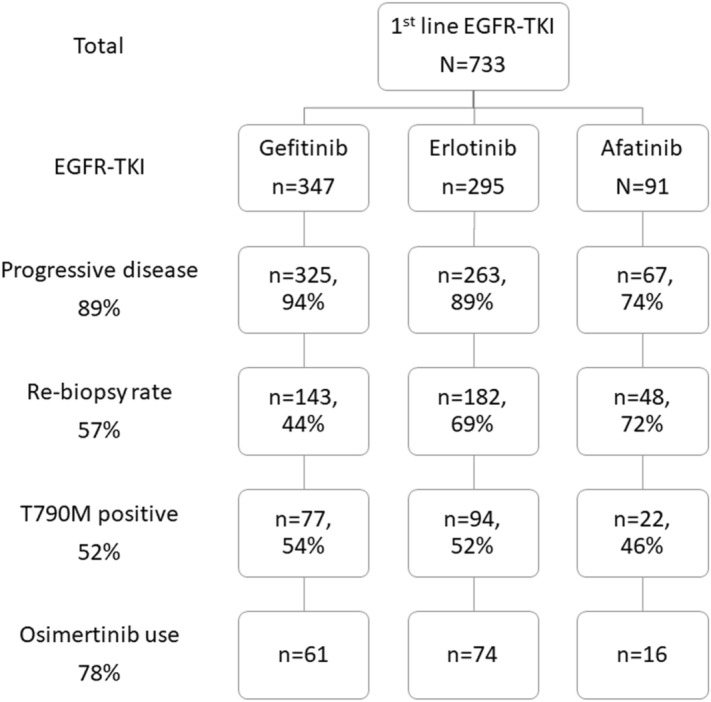
The patient collection flow chart.

In total, 733 advanced *EGFR*-mutant NSCLC patients with first-line EGFR-TKI treatment were enrolled for analysis (Fig. 1). Three hundred and forty-seven patients received gefitinib, 295 patients received erlotinib and 91 patients received afatinib as first-line treatment. The baseline characteristics are shown in Table 1. The percentage of patients older than 65 years and measured with Eastern Cooperative Oncology Group Performance Status (ECOG PS) 2–4 were higher in patients with gefitinib. The percentage of Central Nerve System (CNS) metastasis was higher in patients with erlotinib. The percentage of males and those who had a history of smoking was higher in patients with afatinib. The Objective Response Rate (ORR) was 72.1%, 73.2%, 67.8% in patients with gefitinib, erlotinib and afatinib, respectively. The Disease Control Rate (DCR) was 88.4%, 92.1%, 93.1% in patients with gefitinib, erlotinib and afatinib, respectively. There was no statistical difference in ORR and DCR between the three types of EGFR-TKI.

**Table 1:** The characteristics of *EGFR*-mutant NSCLC patients with first-line EGFR-TKI treatment (n = 733).

Characteristics	First-line EGFR-TKI			P value^a^
	Gefitinib	Erlotinib	Afatinib	
**Age**				< 0.001
< 65	148 (42.7)	171 (58.0)	60 (65.9)	
≥ 65	199 (57.3)	124 (42.0)	31 (34.1)	
**Sex**				< 0.001
Male	115 (33.1)	121 (41.0)	53 (58.2)	
Female	232 (66.9)	174 (59.0)	38 (41.8)	
**Smoking status**				0.003
NS	271 (78.1)	225 (76.3)	55 (60.4)	
C/FS	76 (21.9)	70 (23.7)	36 (39.6)	
**ECOG PS**				0.001
0–1	262 (75.5)	255 (86.4)	79 (86.8)	
2–4	85 (24.5)	40 (13.6)	12 (13.2)	
**CNS metastasis**				< 0.001
No	249 (71.8)	137 (46.4)	55 (60.4)	
Yes	98 (28.2)	158 (53.6)	36 (39.6)	
**Baseline *EGFR* mutation status**				0.145
19Del	152 (43.8)	150 (50.8)	47 (51.6)	
L858R	195 (56.2)	145 (48.2)	44 (48.4)	
Objective response rate	72.1%	73.2%	67.8%	0.614
Disease control rate	88.4%	92.1%	93.1%	0.251

*EGFR* epidermal growth factor receptor, *NSCLC* non-small-cell lung cancer, *TKI* tyrosine kinase inhibitor, *NS* non-smoker, *C/FS* current/former-smoker, *ECOG PS* Eastern Cooperative Oncology Group performance status, *CNS* central nervous system.

^a^By Fisher's exact test.

### The PFS of first-line gefitinib, erlotinib and afatinib treatment (PFS1) (n = 733)

**Figure 2:**
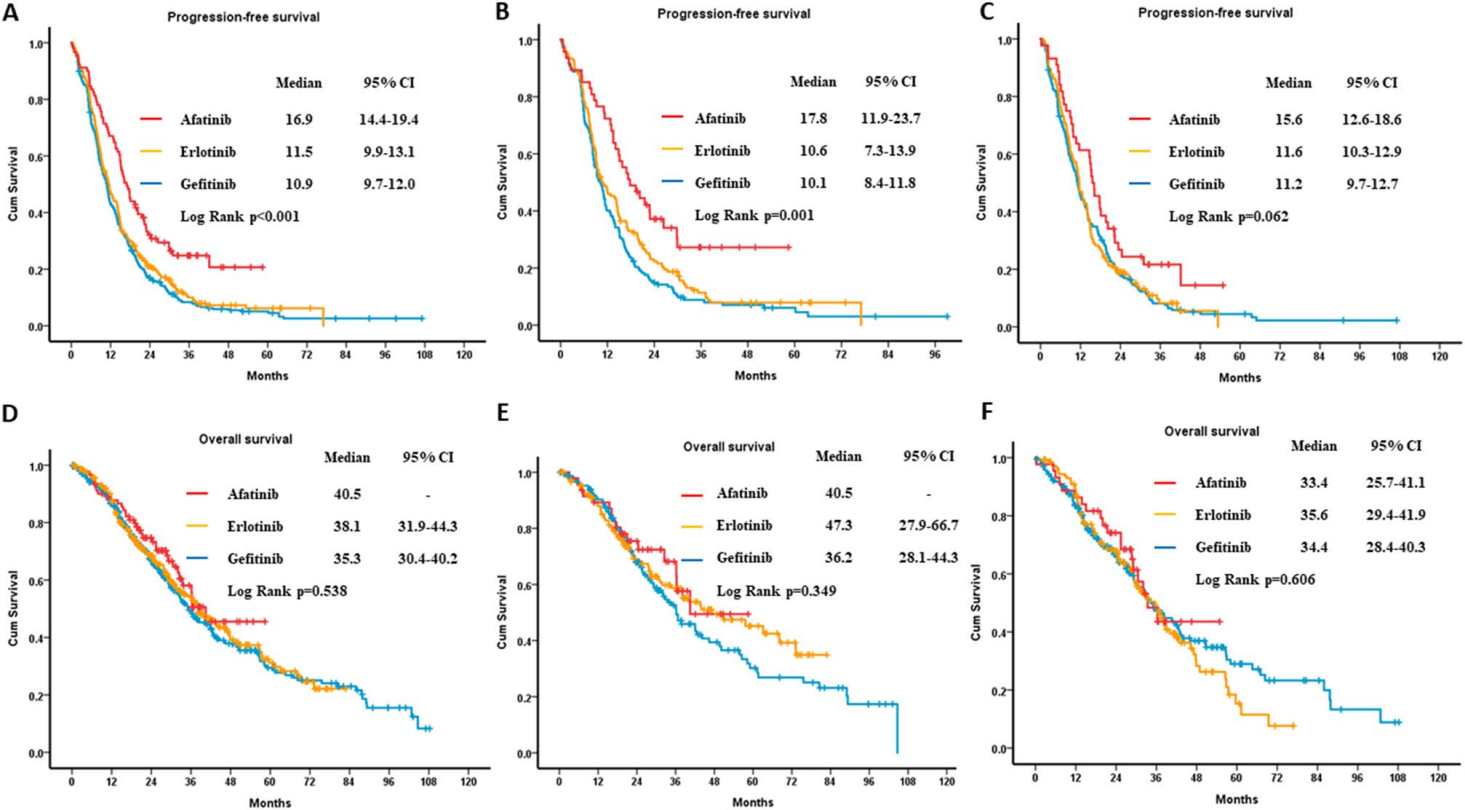
The PFS1 and OS of first-line EGFR-TKI in patients with advanced EGFR-mutant NSCLC. (**A**) The PFS1 of different EGFR-TKIs. (**B**) The PFS1 of different EGFR-TKIs in patients with exon 19 deletion. (**C**) The PFS1 of different EGFR-TKIs in patients with L858R. (**D**) The OS of different EGFR-TKIs. (**E**) The OS of different EGFR-TKIs in patients with exon 19 deletion. (**F**) The OS of different EGFR-TKIs in patients with L858R. *PFS* progression-free survival, *OS* overall survival, *EGFR-TKI* epidermal growth factor receptor–tyrosine kinase inhibitor, *NSCLC* non-small-cell lung cancer.

The estimated median PFS1 of gefitinib, erlotinib and afatinib was 10.9 months (95% CI 9.7–12.0), 11.5 months (95% CI 9.9–13.1), and 16.9 months (95% CI 14.4–19.4) (log rank test, p < 0.001), respectively (Fig. 2A). Regarding different *EGFR* mutation, in patients with exon 19 deletion, the estimated median PFS1 was 10.1 months (95% CI 8.4–11.8) in the gefitinib group, 10.6 months (95% CI 7.3–13.9) in the erlotinib group, and 17.8 months (95% CI 11.9–23.7) in the afatinib group (log rank test, p = 0.001) (Fig. 2B). Alternatively, in patients with exon 21 L858R mutation, the estimated median PFS1 was 11.2 months (95% CI 9.7–12.7) in the gefitinib group, 11.6 months (95% CI 10.3–12.9) in the erlotinib group, and 15.6 months (95% CI 12.6–18.6) in the afatinib group (log rank test, p = 0.062) (Fig. 2C). Univariate and multivariate analysis using the Cox proportional hazard model showed that ECOG PS, CNS metastasis and first-line EGFR-TKIs were statistically significant independent factors for PFS1 (Table 2). Patients given gefitinib (adjusted HR 1.35; 95% CI 1.19–1.53, p < 0.001) and erlotinib (adjusted HR 1.10; 95% CI 0.98–1.25, p = 0.090) as first-line treatment had a higher risk of progressive disease than patients treated with afatinib.

**Table 2:** Univariate and multivariate analysis of progression-free survival in NSCLC patients with first-line EGFR-TKI treatment (PFS1) (n = 733).

Characteristics	HR (95% CI)^a^	P value	Adjusted HR (95% CI)^a^	P value
**Age**				
< 65	Reference			
≥ 65	0.91 (0.78–1.06)	0.204		
**Sex**				
Male	Reference			
Female	0.93 (0.79–1.08)	0.333		
**Smoking status**				
NS	Reference			
C/FS	1.12 (0.93–1.33)	0.227		
**ECOG PS**				
2–4	Reference			
0–1	0.78 (0.71–0.86)	< 0.001	0.78 (0.71–0.86)	< 0.001
**CNS metastasis**				
Yes	Reference			
No	0.88 (0.81–0.95)	0.001	0.88 (0.81–0.95)	< 0.001
**Baseline *EGFR *mutation status**				
L858R	Reference			
19Del	0.97 (0.90–1.05)	0.460		
**1st line EGFR-TKI**				
Afatinib	Reference			
Gefitinib	1.76 (1.36–2.30)	< 0.001	1.35 (1.19–1.53)	< 0.001
Erlotinib	1.59 (1.21–2.07)	0.001	1.10 (0.98–1.25)	0.09

*NSCLC *non-small-cell lung cancer, *EGFR* epidermal growth factor receptor, *TKI* tyrosine kinase inhibitor, *HR* hazard ratio, *CI* confidence interval, *NS* non-smoker, *C/FS* current/former-smoker, *ECOG* *PS* Eastern Cooperative Oncology Group performance status, *CNS* central nervous system.

^a^By Cox proportional hazard model.

### The OS of first-line gefitinib, erlotinib and afatinib treatment

The estimated median OS was 35.3 months (95% CI 30.4–40.2) in the gefitinib group, 38.1 months (95% CI 31.9–44.3) in the erlotinib group, and 40.5 months in the afatinib group (log-rank test, p = 0.538) (Fig. 2D). Concerning different *EGFR* mutation, in patients with exon 19 deletion, the estimated median OS was 36.2 months (95% CI 28.1–44.3) in the gefitinib group, 47.3 months (95% CI 27.9–66.7) in the erlotinib group, and 40.5 months in the afatinib group (log rank test, p = 0.349) (Fig. 2E). Alternatively, in patients with exon 21 L858R mutation, the estimated median OS was 34.4 months (95% CI 28.4–40.3) in the gefitinib group, 35.6 months (95% CI 29.4–41.9) in the erlotinib group, and 33.4 months (95% CI 25.7–41.1) in the afatinib group (log rank test, p = 0.606) (Fig. 2F). According to the Cox proportional hazard model, female gender (adjusted HR 0.70; 95% CI 0.54–0.92, p = 0.009), ECOG PS 0–1 (adjusted HR 0.52; 95% CI 0.40–0.67, p < 0.001), without CNS metastasis (adjusted HR 0.66; 95% CI 0.54–0.81, p < 0.001) and exon 19 deletion mutation (adjusted HR 0.74; 95% CI 0.60–0.91, p = 0.04) were all statistically significant factors to predict better OS (Supplementary Table S1). The different first-line EGFR-TKI use did not influence the outcome of OS.

### Patients’ characteristics of T790M-positive NSCLC patients with osimertinib treatment after progressive disease to first-line EGFR-TKIs

Six hundred and fifty-five patients experienced progressive disease after first-line EGFR-TKIs use (Fig. 1). Three hundred and seventy-three patients received re-biopsies (tissue biopsy, 279 patients; liquid biopsy, 190 patients; both, 96 patients). The total positive rate of T790M was 51.7% (tissue biopsy, 50.5%; liquid biopsy, 41.6%). In our analysis, 96 patients received both tissue and liquid biopsy after progressive disease to first-line EGFR-TKI. Among 47 patients with T790M in tissue biopsy, 18 patients harbored T790M in liquid biopsy. Among 49 patients without T790M in tissue biopsy, 15 patients harbored T790M in liquid biopsy. Additionally, 94 patients received liquid biopsy only (23.1% in gefitinib, 26.4% in erlotinib, and 27.1% in afatinib).

One hundred and fifty-one patients who harbored T790M received osimertinib as subsequent treatment. The patients’ demographic data is listed in Table 3. We divided the 151 patients into three groups according to first-line EGFR-TKI use. The percentage of patients older than 65 years was higher in the gefitinib group, with the percentage of CNS metastasis being higher in patients treated with erlotinib. The ORR and DCR of osimertinib was 56.3% and 88.0%, respectively.

**Table 3:** The characteristics of T790M^+^ NSCLC patients with osimertinib treatment after progressive disease to first-line EGFR-TKIs (n = 151).

Characteristics	First-line EGFR-TKI			P value^a^
	Gefitinib	Erlotinib	Afatinib	
**Age**				0.020
< 65	26 (42.6)	49 (66.2)	10 (62.5)	
≥ 65	35 (57.4)	25 (33.8)	6 (37.5)	
**Sex**				0.387
Male	16 (26.2)	27 (36.5)	6 (37.5)	
Female	45 (73.8)		10 (62.5)	
**Smoking status**				0.695
NS	53 (86.9)	60 (81.1)	13 (81.3)	
C/FS	8 (13.1)	14 (18.9)	3 (18.7)	
**ECOG PS**				0.163
0–1	56 (91.8)	67 (90.5)	12 (75.0)	
2–4	5 (9.2)	7 (9.5)	4 (25.0)	
**CNS metastasis**				< 0.001
No	48 (78.7)	34 (45.9)	11 (68.8)	
Yes	13 (21.3)	40 (54.1)	5 (31.2)	
**Baseline *EGFR* mutation status**				0.945
19Del	33 (54.1)	40 (54.1)	8 (50.0)	
L858R	28 (45.9)	34 (45.9)	8 (50.0)	

*NSCLC* non-small-cell lung cancer, *EGFR* epidermal growth factor receptor, *TKI* tyrosine kinase inhibitor, *NS* non-smoker, *C/FS* current/former-smoker, *ECOG PS* Eastern Cooperative Oncology Group performance status, *CNS* central nervous system.

^a^By Fisher's exact test.

### The PFS and OS of T790M-positive patients with osimertinib use (n = 151)

**Figure 3:**
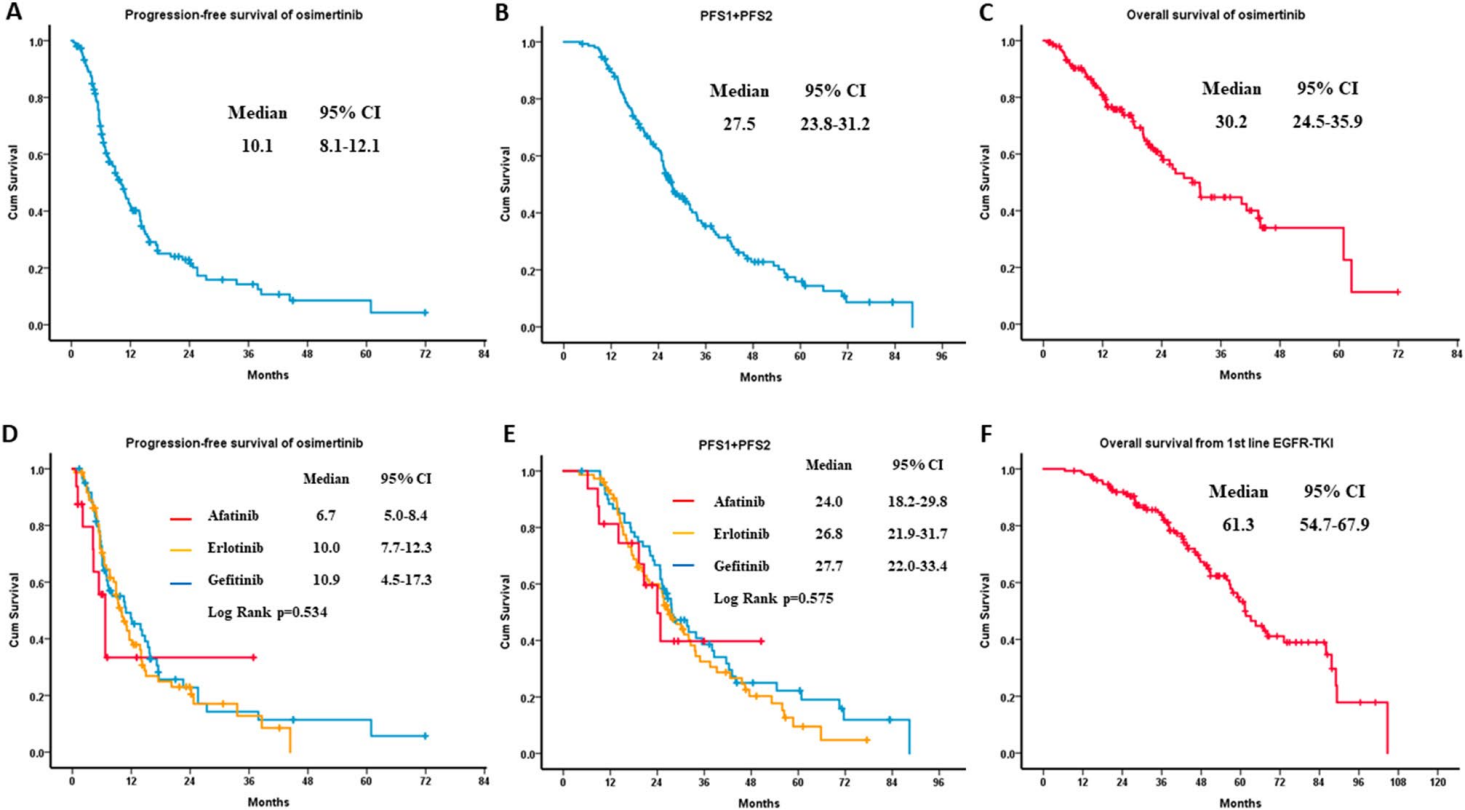
The clinical efficacy of osimertinib in T790M-mutant NSCLC patients with progressive disease to first-line EGFR-TKI. (**A**) The PFS of osimertinib (PFS2) in all patients. (**B**) The PFS1 + PFS2 in all patients. (**C**) The OS of osimertinib in all patients. (**D**) The PFS2 in different first-line EGFR-TKIs. (**E**) The PFS1 + PFS2 in different first-line EGFR-TKIs. (**F**) The OS from first-line EGFR-TKIs. *NSCLC* non-small-cell lung cancer, *EGFR-TKI* epidermal growth factor receptor–tyrosine kinase inhibitor, *PFS* progression-free survival, *OS* overall survival.

The estimated median PFS of first-line EGFR-TKI (PFS1) was 14.0 months (95% CI 11.9–16.1). The estimated median PFS of osimertinib (PFS2) was 10.1 months (95% CI 8.1–12.1) (Fig. 3A). The estimated median PFS1 + PFS2 was 27.5 months (95% CI 23.8–31.2) (Fig. 3B). The estimated median OS of osimertinib was 30.2 months (95% CI 24.5–35.9) (Fig. 3D). The estimated median OS from first-line EGFR-TKI to death was 61.3 months (95% CI 54.7–67.9) (Fig. 3F). Concerning different first-line EGFR-TKIs, the estimated median PFS2 was 10.9 months (95% CI 4.5–17.3) in the gefitinib group, 10.0 months (95% CI 7.7–12.3) in the erlotinib group, and 6.7 months (95% CI 5.0–8.4) in the afatinib group (p = 0.534) (Fig. 3D). In addition, the estimated median PFS1 + PFS2 was 27.7 months (95% CI 22.0–33.4) in the gefitinib group, 26.8 months (95% CI 21.9–31.7) in the erlotinib group, and 24.0 months (95% CI 18.2–29.8) in the afatinib group (p = 0.575) (Fig. 3E). The univariate and multivariate analyses demonstrated that ECOG PS 0–1 (adjusted HR 0.51; 95% CI 0.29–0.90, p = 0.020) was the only statistically significant factor to predict better PFS2 (Table 4), while there was no obvious factor affecting the outcome of PFS1 + PFS2 (Table 5).

**Table 4:** Univariate and multivariate analysis of progression-free survival in T790M^+^ NSCLC patients with osimertinib treatment (PFS2) (n = 151).

Characteristics	HR (95% CI)^a^	P value	Adjusted HR (95% CI)^a^	P value
**Age**				
< 65	Reference			
≥ 65	0.78 (0.53–1.15)	0.213		
**Sex**				
Male	Reference			
Female	0.87 (0.58–1.29)	0.478		
**Smoking status**				
NS	Reference			
C/FS	1.59 (0.95–2.65)	0.079		
**ECOG PS**				
2–4	Reference			
0–1	0.52 (0.30–0.90)	0.019	0.51 (0.29–0.90)	0.020
**CNS metastasis**				
Yes	Reference			
No	0.87 (0.59–1.28)	0.483		
**Baseline *EGFR *mutation statu*s***				
L858R	Reference			
19Del	0.93 (0.63–1.36)	0.709		
**1st line EGFR-TKI**				
Afatinib	Reference			
Gefitinib	0.66 (0.31–1.40)	0.279		
Erlotinib	0.73 (0.35–1.55)	0.416		

*NSCLC *non-small-cell lung cancer, *HR* hazard ratio, *CI* confidence interval, *NS* non-smoker, *C/FS* current/former-smoker, *ECOG PS* Eastern Cooperative Oncology Group performance status, *CNS* central nervous system, *EGFR* epidermal growth factor receptor, *TKI* tyrosine kinase inhibitor.

^a^By Cox proportional hazard model.

**Table 5:** Univariate and multivariate analysis of PFS1^a^ + PFS2^b^ in T790M + NSCLC patients (n = 151).

Characteristics	HR (95% CI)^c^	P value	Adjusted HR (95% CI)^c^	P value
**Age**				
< 65	Reference			
≥ 65	0.68 (0.46–1.00)	0.051	0.66 (0.42–1.02)	0.059
**Gender**				
Male	Reference			
Female	0.89 (0.60–1.33)	0.563		
**Smoking status**				
NS	Reference			
C/FS	1.42 (0.86–2.36)	0.175		
**ECOG PS**				
2–4	Reference			
0–1	0.58 (0.34–1.00)	0.051	0.63 (0.36–1.11)	0.109
**CNS metastasis**				
Yes	Reference			
No	0.70 (0.47–1.04)	0.078		
**Baseline *EGFR* mutation status**				
L858R	Reference			
19Del	0.94 (0.64–1.37)	0.734		
**1st line EGFR**-TKI				
Gefitinib	Reference			
Erlotinib	1.23 (0.83–1.83)	0.312		
Afatinib	1.25 (0.58–2.67)	0.570		

*PFS *progression-free survival, *NSCLC* non-small-cell lung cancer, *HR* hazard ratio, *CI* confidence interval, *NS* non-smoker, *C/FS* current/former-smoker, *ECOG PS* Eastern Cooperative Oncology Group performance status, *CNS* central nervous system, *EGFR* epidermal growth factor receptor, *TKI* tyrosine kinase inhibitor.

^a^PFS1, progression-free survival of first-line EGFR-TKI.

^b^PFS2, progression-free survival of osimertinib.

^c^By Cox proportional hazard model.

## Discussion

Our research has demonstrated that advanced *EGFR*-mutant NSCLC patients with first-line afatinib treatment displayed better PFS than patients with gefitinib or erlotinib use (PFS1). The present study has also proved the real-world efficacy of osimertinib in *EGFR*-mutant NSCLC patients with progressive disease to first-line EGFR-TKIs who harbored T790M after rebiopsy. The PFS of osimertinib (PFS2) and PFS1 + PFS2 was not influenced by different first-line EGFR-TKI use. Additionally, the median OS reached up to 5 years in patients with first-line EGFR-TKI who had undergone subsequent osimertinib treatment.

In previous real-world studies, the difference in clinical efficacy between first- and second-generation EGFR-TKIs was not conclusive. Kuan et al*.* found that the PFS of erlotinib and afatinib as first-line EGFR-TKI treatments in advanced *EGFR*-mutant NSCLC patients was longer than that resulting from gefitinib^17^. Tu et al*.* stated that patients who were administered afatinib as first-line treatment experienced better PFS than patients given gefitinib (median 12.2 months versus 9.8 months, p = 0.035), while there was no difference in PFS between erlotinib and afatinib (median 11.4 months versus 12.2 months, p = 0.38)^18^. Lin et al*.* demonstrated that PFS was the same between gefitinib, gefitinib and afatinib (median 12.4 months versus 14.4 months versus 12.4 months)^19^. In Kim’s research, the PFS of afatinib was significantly longer than that of both gefitinib and erlotinib (median 19.1 months versus 13.7 months versus 14.0 months, p = 0.001), however the OS of EGFR-TKIs did not differ^20^.

In our study, the median PFS1 of afatinib was 16.9 months, gefitinib 10.9 months and erlotinib 11.5 months (log rank test, p < 0.001). Patients with gefitinib as first-line EGFR-TKI treatment were older and worse ECOG PS than patients with erlotinib and afatinib. The different patients’ characteristics could result in the shorter PFS in patients with gefitinib treatment. Although the baseline demographic data was not equal between the different EGFR-TKIs, according to multivariate analysis, we discovered that patients given gefitinib as a first-line treatment still had a statistically significant 35% higher risk of progressive disease than patients who were administered afatinib (adjusted HR 1.35; 95% CI 1.19–1.53, p < 0.001). Additionally, there was a trend towards PFS1 being better with afatinib than erlotinib (adjusted HR 1.10; 95% CI 0.98–1.25, p = 0.090). First-generation EGFR-TKIs bind to EGFR receptors reversibly, while afatinib irreversibly blocks the signal of the pan-Erb B family of receptors^21^. In vitro, the IC_50_ (50% inhibitory concentration) of afatinib was lower than the IC_50_ of gefitinib and erlotinib against *EGFR*-mutant cell lines, both in exon 19 deletion and exon 21 L858R point mutation^22^. The different potency and mechanisms of EGFR-TKIs may result in different clinical efficacies.

In the present study, 89% of patients experienced progressive disease after first-line EGFR-TKI use, with 57% of patients receiving tissue or liquid re-biopsy. The frequency of T790M was 51.7%, and the positive rate of T790M was not different between first-line EGFR-TKI treatment with gefitinib, erlotinib or afatinib (53.8% versus 51.6% versus 45.8%, p = 0.632). The results were consistent with previous studies^14,15^. In a clinical trial of AURA3, when patients who harbored T790M after drug resistance to first-line EGFR-TKI received osimertinib treatment, the median PFS was 10.1 months and the median OS was 26.8 months^16,23^. On the other hand, the global real-world study of ASTRIS presented that the ORR of osimertinib was 57.1% and median PFS was 11.1 months for Osimertinib^24^. According to a GioTag study, the median time on treatment with afatinib and osimertinib was 27.7 months, while the median OS was 37.6 months in patients with *EGFR*-mutation-positive NSCLC^25^.

In our investigation, 151 T790M^+^ NSCLC patients with an acquired resistance to first-line EGFR-TKIs then received osimertinib as subsequent treatment. The ORR and DCR of osimertinib was 56.3% and 88.0%, respectively. The median PFS2 was 10.1 months, OS of osimertinib was 30.2 months, median PFS1 + PFS2 was 27.5 months, and the median OS from first-line EGFR-TKI to death was 61.3 months. Our findings were consistent with previous clinical trials and real-world analysis, and we also confirmed the clinical efficacy of osimertinib in acquired T790M-mutant NSCLC patients who had progressive disease to first-line EGFR-TKI. Additionally, our study demonstrated that not only afatinib, but also gefitinib and erlotinib, can provide optimal clinical outcomes if the patients with secondary T790M mutation could receive subsequent osimertinib treatment. Although the PFS1 and PFS1 + PFS2 were relatively shorter in the afatinib group than in the first-generation TKI groups, the result did not reach a statistically significant level. Additionally, there were only 16 patients who were administered afatinib as first-line treatment who received subsequent osimertinib use. Thus, it was difficult to draw a conclusion on the impact which different first-line EGFR-TKIs have on the clinical outcome of sequential osimertinib treatment.

Compared with other real-world studies, we included higher patient numbers, and our patient group was relatively homogenous. We only enrolled patients with exon 19 deletion or exon 21 L858R point mutation. In the analysis of the efficacy of different first-line EGFR-TKIs, our data was relative mature due to nearly 90% of the patients experiencing progressive disease to first-line EGFR-TKIs. Additionally, we discussed the impact of different first-line EGFR-TKI use to the clinical outcome of sequential osimertinib treatment. There has been a few papers which have discussed this issue. However, our study did have some limitations. The present research was a retrospective, single center study, where only Taiwanese were eligible for analysis. Thus, more bias may have been present when compared with other studies which had been prospectively designed. Additionally, our findings may not be generalizable for other ethnic populations. Furthermore, we did not analyze the effects of the combination treatment with anti-angiogenic agents, the treatment modality between first-line EGFR-TKIs and osimertinib, or the duration between progressive disease with first-line EGFR-TKIs and the start time of sequential osimertinib use. In Taiwan, gefitinib was reimbursed earlier than erlotinib and afatinib, and the AURA3 study was published in 2017^16^. However, we enrolled patients from 2011 in present research. Above reasons explained the phenomenon that the rebiopsy rate in patients with gefitinib as first-line treatment was relatively lower than patient with erlotinib and afatinib. Otherwise, since osimertinib was reimbursed since April 2020 in Taiwan, some patients did not receive osimertinib treatment owing to the economic reason. Finally, a relatively small number of patients taking afatinib as first-line treatment were included in our cohort, and this may affect the final data analysis, so we should therefore interpret the results carefully.

Our findings shed light on the clinical efficacy of different first-line EGFR-TKIs in advanced *EGFR*-mutant NSCLC patients, the clinical efficacy of sequential osimertinib therapy in patients with T790M-positive after acquired resistance to first-line EGFR-TKI, as well as the impact of different first-line EGFR-TKI use to the clinical outcomes of subsequent osimertinib treatment. In conclusion, our research has demonstrated that advanced *EGFR*-mutant NSCLC patients using afatinib as first-line EGFR-TKI treatment experienced significant longer PFS than patients treated with gefitinib, while also showing better results than patients treated with erlotinib. After the patients experienced progressive disease to first-line EGFR-TKIs, sequential osimertinib treatment in patients with T790M proved to be both effective and satisfying not only in patients given afatinib but also in patients given gefitinib and erlotinib as first-line EGFR-TKIs therapy. Finally, patients administered first-line gefitinib, erlotinib and afatinib, followed by osimertinib treatment, can have a promising median OS more than 5 years.

## Methods

### Study design and patients

This study was a retrospective, single-center, observational study at Taichung Veterans General Hospital (TCVGH) in Taiwan. The study was conducted ethically in accordance with the World Medical Association Declaration of Helsinki and was approved by the Institutional Review Board (IRB) of TCVGH, Taiwan, and written informed consent documents for genetic testing and clinical data records were obtained from all patients (IRB No. CF12019).

We enrolled *EGFR*-mutant NSCLC patients between June 2011 and December 2018. To be eligible for the study, patients had to fulfill the following inclusion criteria: a diagnosis of histologically and cytologically confirmed NSCLC, recurrent or inoperable advanced stage IIIB to stage IV lung cancer according to the 7th edition of the American Joint Committee for Cancer (AJCC) staging system^26^, activating *EGFR* mutation with exon 19 deletion or exon 21 L858R point mutation, as well as treatment with first-line, first- or second-generation EGFR-TKIs, including gefitnib, erlotinib and afatinib. Patients were excluded if they had *EGFR* mutations with complex mutations, or if they had been diagnosed with another malignancy under treatment. Computed tomography of the chest was performed every 3 months for National Health Insurance reimbursement. The treatment response to EGFR-TKIs was evaluated by the Response Evaluation Criteria in Solid Tumors (Version 1.1)^27^.

Each patient’s demographic and clinical data, including age, sex, smoking status, ECOG PS, condition of their CNS metastasis, baseline *EGFR* mutation status, type of EGFR-TKI treatment, and their response to EGFR-TKI, PFS and OS (from EGFR-TKI use to death) of EGFR-TKI were collected for analysis. Patients who had progressive disease to first-line EGFR-TKIs and had undergone a tissue or liquid biopsy with T790M-positive and received osimertinib treatment were included, in order to analyze the clinical efficacy of third-generation EGFR-TKI. We defined PFS1 as the time from the first dose of first-line EGFR-TKI to progression or death, while the definition of PFS2 was determined as the time from the first dose of osimertinib to progression or death.

### EGFR mutation test for tumor tissue and liquid biopsy

*EGFR* mutations test for tumor tissue and liquid biopsy were based on our previous studies^4,28–30^. For tumor tissue detection, the detection procedure was according to the user’s manual of the MassARRAY^®^ System (Cat. No.10411, SEQUENIM, San Diego, CA acquired by Agena Bioscience, http://agenabio.com/, San Diego, CA at 2014). Extracted DNA was performed serial biochemical reactions including 40 cycles PCR reaction; SAP (shrimp alkaline phosphatase) treatment and 200 cycles signal nucleotide extension reaction by using iPLEX Pro^®^ reagent kit containing Sequenase, iPLEX Pro^®^ reaction mixture, and home-designed probes. After SpectroClean Resin clean up, samples were loaded onto the matrix of SpectroCHIP by Nanodispenser (Matrix) then analyzed by Bruker Autoflex MALDI-TOF MS. Data were collected and analyzed by MassARRAY Typer (version 4) software (Agena Bioscience). For plasma cell-free detection, the combination of peptide nucleic acid (PNA) specific for wild-type allele to block amplification and MALDI-TOF MS method was utilized according to the method used in tumor biopsy with some modification. PNA were synthesized by PanaGene (Daejeon, Korea). The final optimized concentration of PNA to efficiently block wild-type alleles was 25 μM in PCR reactions. All tests were performed in the ISO15189-certified clinical center laboratory of National Center of Excellence for Clinical Trial and Research of the National Taiwan University Hospital.

### Statistical analyses

We used the Fisher’s exact test to assess the difference in patients’ characteristics between gefitinib, erlotinib and afatinib. Survival curves, including PFS and OS, were estimated using the Kaplan–Meier method. Differences in survival time were analyzed by the log-rank test. A Cox proportional hazard model was performed to evaluate the factors associated with PFS and OS in both univariate and multivariate analysis. All statistical tests were done with SPSS23.0 (SPSS Inc., Chicago, IL, USA). Two-tailed tests and P values < 0.05 for significance were used.

## Supplementary Information

Supplementary Information 1.

## Data Availability

All data needed to evaluate the conclusions in the paper are present in the paper or the Supplementary Materials.
