# Publisher Correction: The impact of different first-line EGFR-TKIs on the clinical outcome of sequential osimertinib treatment in advanced NSCLC with secondary T790M

**DOI:** 10.1038/s41598-021-97248-w

**Published:** 2021-08-30

**Authors:** Yen-Hsiang Huang, Jeng-Sen Tseng, Kuo-Hsuan Hsu, Kun-Chieh Chen, Kang-Yi Su, Sung-Liang Yu, Jeremy J. W. Chen, Tsung-Ying Yang, Gee-Chen Chang

**Affiliations:** 1 Division of Chest Medicine, Department of Internal Medicine, Taichung Veterans General Hospital, No. 1650, Sect. 4, Taiwan Boulevard, Taichung, 407 Taiwan; 2 Institute of Biomedical Sciences, National Chung Hsing University, No. 145, Xingda Rd., South Dist., Taichung, 402 Taiwan; 3 Faculty of Medicine, School of Medicine, National Yang-Ming University, No. 155, Sect. 2, Linong St., Taipei, 112 Taiwan; 4 Division of Critical Care and Respiratory Therapy, Department of Internal Medicine, Taichung Veterans General Hospital, No. 1650, Sect. 4, Taiwan Boulevard, Taichung, 407 Taiwan; 5 Division of Pulmonary Medicine, Department of Internal Medicine, Chung Shan Medical University Hospital, No.110, Sec. 1, Jianguo N. Road, Taichung, 402 Taiwan; 6 Institute of Medicine, Chung Shan Medical University, No.110, Sec. 1, Jianguo N. Road, Taichung, 402 Taiwan; 7 School of Medicine, Chung Shan Medical University, No. 110, Sec. 1, Jianguo N. Road, Taichung, 402 Taiwan; 8 Department of Clinical Laboratory Sciences and Medical Biotechnology, College of Medicine, National Taiwan University, No. 1, Sect. 1, Jen Ai Road, Taipei, 100 Taiwan; 9 Department of Laboratory Medicine, National Taiwan University Hospital, No. 7, Zhung-Shan South Road, Taipei, 100 Taiwan; 10 Graduate Institute of Clinical Medicine, College of Medicine, National Taiwan University, No. 1, Sect. 1, Jen Ai Road, Taipei, 100 Taiwan; 11 Center of Genomic and Precision Medicine, National Taiwan University, No. 2, Syu-jhou Road, Taipei, 100 Taiwan; 12 Institute of Medical Device and Imaging, College of Medicine, National Taiwan University, No. 1, Sect. 1, Jen Ai Road, Taipei, 100 Taiwan; 13 Graduate Institute of Pathology, College of Medicine, National Taiwan University, No. 1, Sect. 1, Jen Ai Road, Taipei, 100 Taiwan

Correction to: *Scientific Reports* 10.1038/s41598-021-91657-7, published online 08 June 2021

The original version of this Article contained errors in Table 4, where the P value was omitted and the “Adjusted HR (95% CI)^a^” value for “ECOG PS” was incorrectly assigned. The correct and incorrect values appear below.

Incorrect:

**Table:** 

Characteristics	HR (95% CI)^a^	P value	Adjusted HR (95% CI)^a^	P value
**Smoking status**				
NS	Reference		0.51 (0.29–0.90)	
C/FS	1.59 (0.95–2.65)	0.079		
**ECOG PS**				
2–4	Reference			
0–1	0.52 (0.30–0.90)	0.019		

Correct:

**Table:** 

Characteristics	HR (95% CI)^a^	P value	Adjusted HR (95% CI)^a^	P value
**Smoking status**				
NS	Reference			
C/FS	1.59 (0.95–2.65)	0.079		
**ECOG PS**				
2–4	Reference			
0–1	0.52 (0.30–0.90)	0.019	0.51 (0.29–0.90)	0.020

The original Article has been corrected.

