# Supplementary material for: The impact of different first-line EGFR-TKIs on the clinical outcome of sequential osimertinib treatment in advanced NSCLC with secondary T790M
Source: Sci Rep. 2021 Jun 8;11:12084. doi: 10.1038/s41598-021-91657-7 (PMC8187359; doi:10.1038/s41598-021-91657-7)
Supplement: Supplementary file 1 — Supplementary Information 1. [file 41598_2021_91657_MOESM1_ESM.docx]

Supplementary Table 1. Univariate and multivariate analysis of overall survival in NSCLC patients with first-line EGFR-TKI treatment (n=733)

| Characteristics | HR (95% CI)^a^ | P value | Adjusted HR (95% CI)^a^ | P value |
| --- | --- | --- | --- | --- |
| Age  <65  ≥65  Gender  Male  Female  Smoking status  NS  C/FS  ECOG PS  2-4  0-1  CNS metastasis  Yes  No  Baseline *EGFR* mutation status  L858R  19Del  1^st^ line EGFR-TKI  Afatinib  Gefitinib  Erlotinib | Reference  1.05 (0.86-1.29)  Reference  0.71 (0.56-0.87)    Reference  1.41 (1.12-1.18)  Reference  0.72 (0.64-0.82)  Reference  0.81 (0.73-0.89)  Reference  0.88 (0.79-0.97)  Reference  1.09 (0.93-1.27)  1.04 (0.88-1.22) | 0.621  0.001  0.004    <0.001  <0.001  0.013  0.294  0.659 | 0.70 (0.54-0.92)  1.16 (0.86-1.55)  0.52 (0.40-0.67)  0.66 (0.54-0.81)  0.74 (0.60-0.91) | 0.009  0.336  <0.001  <0.001  0.004 |

Abbreviations: NSCLC, non-small-cell lung cancer; EGFR, epidermal growth factor receptor; TKI, tyrosine kinase inhibitor; HR, hazard ratio; CI, confidence interval; NS, non-smoker; C/FS, current/former-smoker; ECOG PS, Eastern Cooperative Oncology Group performance status; CNS, central nervous system.

^a^By Cox proportional hazard model.
